# Plant communities converge to resource-dependent transient states during succession on old fields

**DOI:** 10.1038/s41598-025-16501-8

**Published:** 2025-08-23

**Authors:** Jutta Stadler, Roland Brandl, Stefan Klotz

**Affiliations:** 1 Department of Community Ecology, Helmholtz Centre for Environmental Research-UFZ, Theodor-Lieser-Str. 4, 06120 Halle (Saale), Germany; 2 Faculty of Biology, Department of Ecology, Philipps-Universität Marburg, Karl-von-Frisch Str 8, 35032 Marburg, Germany

**Keywords:** Community composition, Fertilisation, Indicator species, Trajectory analysis, Transient stable states, Ecology, Ecology

## Abstract

The community composition of plants during succession may follow trajectories driven by the resource-dependent coexistence of plant species. This predicts the convergence of trajectories to resource-dependent transient or stable states. Here, we report the analyses of yearly vegetation surveys between 1987 and 2022 from two pairs of experimental plots on which agricultural use ceased in 1986. On one plot of each pair, nutrients (N, P, K) were added in an amount similar to the Park Grass Experiment at Rothamsted. We found a decrease in species richness with ongoing secondary succession. After 15 years of succession, species richness levelled off and was ≈ 50% lower for surveys on the fertilised plots. An indicator species analysis found five species characterising the fertilised plots and almost 30 species characterising the non-fertilised plots. As expected, the mean N Ellenberg indicator values of the species typical for fertilised plots were higher (mean 6.3) than for those typical for non-fertilised plots (mean 4.4). Furthermore, the stable state was transient, as indicated by the increase in the distance of plots from their respective medians of the ordination scores after an additional 10-year period.

**Supplementary Information:**

The online version contains supplementary material available at 10.1038/s41598-025-16501-8.

## Introduction

Global change and associated disturbance events affect population dynamics, distribution and community composition of plants and animals^1^. In particular, disturbances (e.g. due to severe weather events) lead to changes in plant and animal communities^2^. The interpretations of the available information often lack consistency due to the system’s long response time^3^. Nevertheless, predicting trajectories of succession after such disturbances is essential for developing strategies to mitigate adverse effects^4^. Thus, we need succession experiments using systems with comparatively short response times. However, even demonstrating a stable state for such communities needs decades-long data^2^.

Succession is a central concept in ecology, describing directional changes in community composition^5–7^. Such changes in community composition occur, if a sterile substrate offers new opportunities for colonisation by plants and animals (primary succession^8,9^. This type of succession differs from secondary succession, which refers to community change following disturbances that produce no sterile substrate^9,10^. Typical examples are the succession after windthrow in forests, clear-cuttings^11,12^ or the succession on old fields^13–15^. The main driver of community composition during primary succession is the arrival of colonizers from outside the area. In contrast, during secondary succession, we have a mixture of surviving plants, arrivals from outside and the establishment of plant species from the seed bank.

A concept intimately linked to succession is the climax^16^. Climax is the final stable state of an ecosystem, determined by the environmental factors of a particular area. Although a majority of plant ecologists have abandoned this concept, it remains a helpful reference point that predicts the convergence of communities to similar species compositions, despite different starting points^7^. If such stable states exist, one further question is which factors determine these states. Resource-based concepts of community composition predict that nutrients are among the primary drivers of the coexistence of plant species^17–19^. Thus, assuming that species of a pool can arrive at two areas with similar nutrient supply, the plant communities of these two areas should converge to the same set of coexisting species despite different starting points. Other concepts invoke plant traits to predict community structure^20,21^ or invoke factors like arrival order, niche pre-emption and priority effects^22–26^. Particularly, the latter processes predict diverging trajectories even for areas with a similar nutrient supply.

Here, we use a long-term experiment on the dynamics in plant communities after the abandonment of agricultural use in 1986. The experiment runs for more than 35 years, sufficiently long to evaluate the occurrence of stable states in plant communities that start with herbaceous plant species^2^. The four plots started from two different initial stages: Two plots were used for crop farming, and two plots as grassland. Furthermore, we fertilised one plot of each pair. Thus, we have two plots differing in nutrient supply for plants. Following the hypotheses of resource-based species coexistence, we predict that (1) succession leads to states with alternative species composition depending on nutrient supply that is stable for a certain period. (2) Irrespective of the plots’ historical legacy, the plant communities converge to a similar species composition depending on nutrient supply. (3) Indicator species for the fertilised and non-fertilised systems should differ according to their N indicator values, with, on average, larger N indicator values in species typical for fertilised plots.

## Materials and methods

The four plots were located near Zöberitz (Halle, Germany; N 51°30´, E 12°1´). The agricultural use ended in 1986 and treatments as well as vegetation surveys started in 1987. We used the vegetation data between 1987 and 2022 for the present analyses. Thus, the experiment spans a 36-year period. Each plot has a dimension of 20 m × 20 m. The plots are paired with each pair on a different land-use system before the experiment: farmland and grassland. We refer to the differences in the background as land-use legacy. Note that this factor is not replicated and used during our statistical analyses to correct environmental differences between the two pairs. The soils of all four plots are nutrient-rich black cotton soil (Chernozem^27^.

The mean yearly temperature of the area near Zöberitz was between 1934 and 2024 around 9.4 °C with a mean precipitation of 534 mm (station Leipzig Schkeuditz: N 51°26´ E 14° 14´). The low precipitation is due to the wind shadow of the Harz mountains, and the climate might be characterised as semi-arid. The mean annual temperature during the experiment (1987 to 2022) showed a clear tendency to become warmer during the observational period (*r* = 0.49, *P* = 0.002; see Supplementary information Fig. S1). Between1987 and 2022 annual precipitation fluctuated around a mean of 525 mm (Fig. S1). The annual precipitation in Germany is between 500 and 2000 mm^28^.

One plot of each pair was fertilised each year using the following fertilizers: calcium ammonium nitrate with 27% N: 120 kg N ha^− 1^ split into two parts with 80 kg ha^− 1^ applied to the plot around May 10th and 40 kg ha^− 1^ around June 06th, superphosphate (45% P) with 40 kg P ha^− 1^ and potash (60% K_2_0) with 100 kg K ha^− 1^ around 10th October^27^. These nutrient additions were of the same order of magnitude as in other experiments analysing the effect of nutrients on vegetation dynamics^29–31^.

Each plot was divided into a grid of 100 subplots measuring four square meters. For each of these subplots, the cover/abundance of the occurring plant species was estimated using the Braun-Blanquet scale (for additional details and analyses of the succession during the first years of this experiment, see^27,32^. For our analyses, we transformed these estimates into a rank scale: *r* = 1, + = 2, 1 = 3 and so on. This rank scale mimics log-transformed abundance estimates^15,33^. Overall, the data allowed for two ways of estimating the abundance of recorded species on each plot. The first possibility was an occupancy measure counting the subplots in which a species was recorded. A second possibility was to average the cover/abundance data across all subplots. Both measures yield similar results, and for the present analysis, we report the averaged cover/abundance data. The latter measure has the advantage that averaging the rank data across 100 subplots results in an estimate of the mean cover-abundance of each species that approaches, according to the central limit theorem, a normal distribution.

A detrended correspondence analysis of the plot × plant species matrix showed a gradient length of 3.5. For gradient lengths between 3 and 4, unimodal and linear analyses should lead to similar results. We decided to use linear methods with standardised data using the Hellinger transformation to reduce the influence of dominant species on the distances between the vegetation surveys^34^. For the transformation, we used the function *decostand* in the R package *vegan*^35^. Subsequently, we used the chord distance to calculate a distance matrix between yearly surveys (using the Hellinger distance in *vegdist*. 2^1/2^ is the upper limit of these distances^35^. However, Procrustes analysis of the configuration of the scores along the first two axes revealed a close correlation between various methods for handling the plot × plant species matrix. We performed a series of analyses with the (transformed) matrix:

1. We calculated the total number of plant species recorded on each of the four plots for each year, further on referred to as species richness. As an initial indication of a stable state we tested for temporal trends of species richness across years using Pearson correlation coefficients. The species richness shows considerable dynamics during succession. Therefore, we selected a time span by eye that showed no apparent trend in species richness. Note that this simple test does not necessarily indicate a stable state as there may be a temporal turnover of species. Therefore, we performed some additional tests.
2. We calculated for each year the percentage of variance in the plot × plant species matrix of Hellinger-transformed plant cover-abundance data accounted for by the legacy of the land use and the nutrient treatment. For these calculations, we used the function *varpart* in *vegan*. We estimated the variance explained by nutrient treatment and legacy for each year using the non-adjusted R². We repeated the analysis of each year. Finally, we plotted R² against the years to provide a visual impression of the variance explained by the nutrient treatment. We expected that the variance explained by the nutrient treatment would increase over time, reaching a maximum value. We used this analysis for descriptive purposes and, based on the few number of plots, report no formal statistical test of the R² values.
3. In a next step, we used trajectory analysis to visualise and test whether communities on the plots showed convergence or divergence depending on the nutrient treatment. For the fertilised as well as non-fertilised plots, we expected convergence independent of the land-use legacy. In contrast, the communities differing in the treatment should show a diverging species composition. For a visual inspection, we plotted the scores of each plot using the first two axes of a principal coordinate analysis of the distance matrix connecting consecutive years. Second, for more formal analysis, we tested whether the distance between two trajectories decreased (convergence) or increased with time using the Mann-Kendall test provided by the function *trajectoryConvergence* in the R-package *ecotraj*^36^. Finally, we used the function *trajectoryAngles* in the package *ecotraj* to characterise the angles between consecutive vegetation samples of each plot. If these variations in the directionality of the trajectory are random, these angles should not be correlated between plots.
4. For another possibility to evaluate whether communities reached a stable state during succession we calculated each year’s distance from the respective median of the four plots^2^ using the function *betadispers* in *vegan*. During stable states this distance should be small and constant across years. If communities diverge from such a stable state one expects that the distance from the median increases with the years.
5. Finally, we performed an indicator species analysis for periods with a stable state using the function *multipatt* from the package *indicspecies*^37^. This is a further possibility for testing whether divergence of communities is an effect of the nutrient treatment. We calculated an indicator value that allows to select the species associated with one of the two treatments (fertilised and non-fertilised). We also report permutation tests to determine the significance of these associations. However, these tests are not strictly valid because of the autocorrelation of the vegetation across years. If the two lists of species differ and if nutrients are the driving force of divergence, we expect that the species characterising fertilised plots have larger nutrient requirements. We extracted Ellenberg N indicator values from the *BiolFlor* data bank^38^ and calculated the mean across the species that characterized fertilised and non-fertilised plots. We expected that the mean N indicator values of these indicator species is higher for fertilised plots compared to the non-fertilised plots.

## Results

**Fig. 1:**
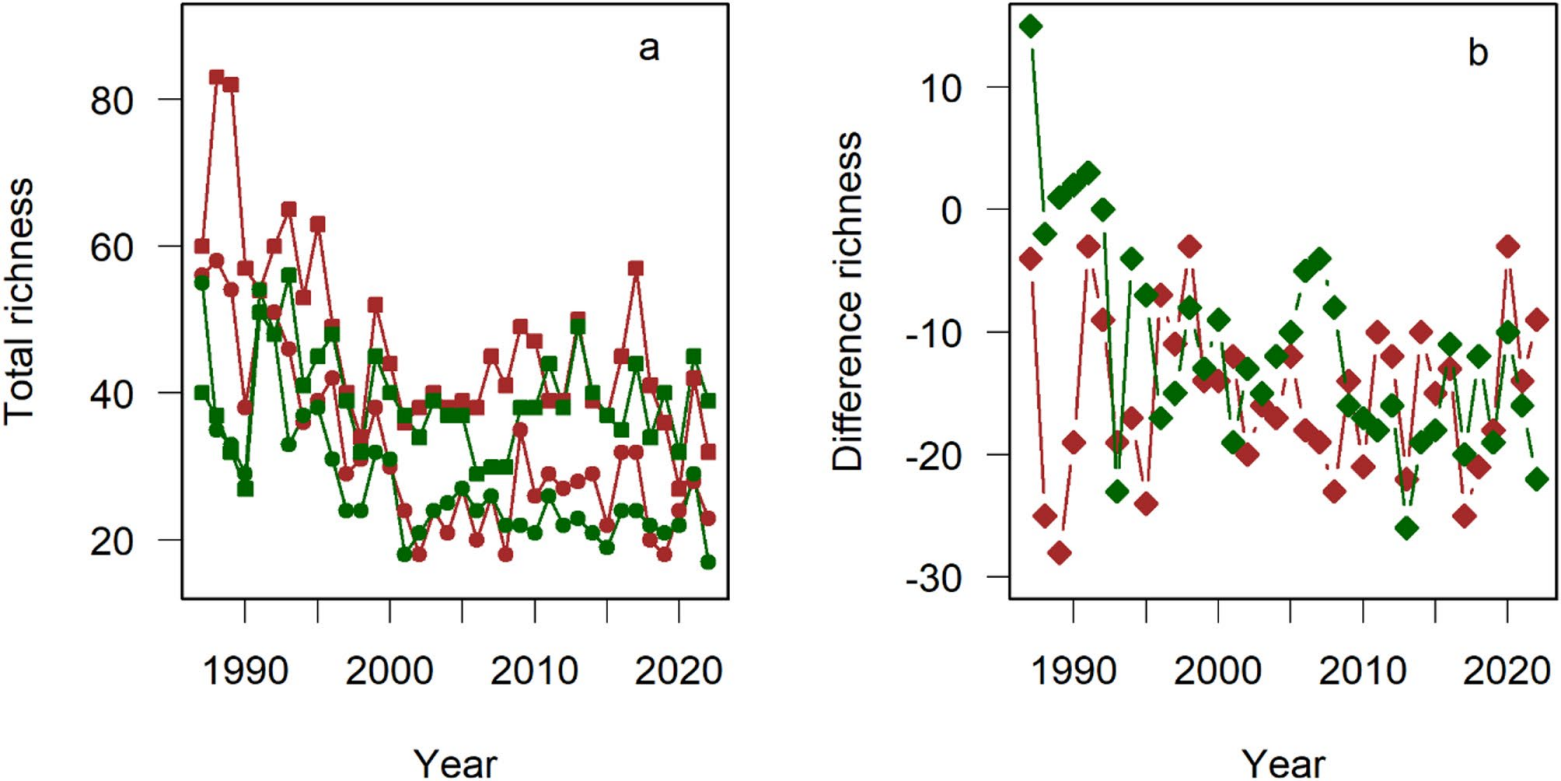
(**a**) Change of the total species richness across the 100 four m² subplots. Legacy of plots: brown farmland, dark green grassland. Circles indicate values of species richness for releveés on fertilised plots and squares on non-fertilised plots. (**b**) Difference between fertilised and non-fertilised plots within each year for farm- (brown) and grassland (dark green). Note the decrease in species richness after the abandonment of the plots for agricultural use in 1986. After the year 2000, the decline levelled off with species richness values depending on the nutrient treatment of the plots. However, die difference decreased across time only for the difference between the two grassland plots (see also Table 1).

The general patterns of succession on our plots matched the well-known trajectories of plant succession on old field. We found a decrease in species richness as well as cover-abundance with time for short lived and the opposite trend for long-lived species (Supplementary information Fig. S2). Species richness decreased with the ongoing succession (Fig. 1a). After 10 to 15 years, species richness levelled off and we found no significant trends in species richness across years for the period 2001 to 2022 (Table 1). However, total species richness on non-fertilised plots was almost twice as high as the species richness on fertilised plots (Fig. 1; Table 1). However, this difference was for the farmland plots a legacy of the situation at the beginning of the experiment (Fig. 1b). For the farmland plots we found that the two plots had already at the beginning of the experiment differing values of species richness and this difference showed in contrast to the grassland plots no decrease with time.

**Fig. 2:**
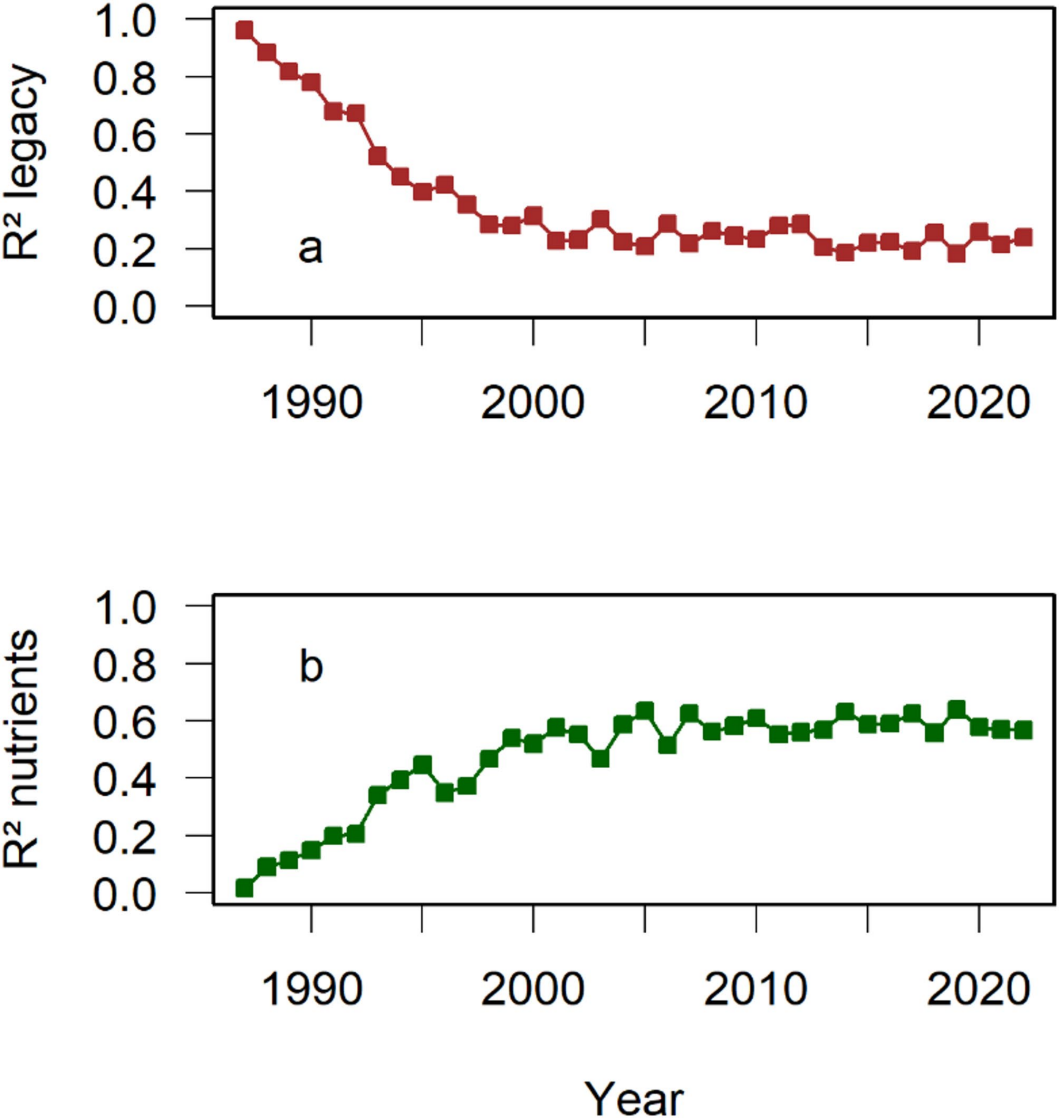
Variation in community composition (R²) explained by the legacy of land use and fertilisation between 1987 and 2022. The explained variation by the legacy dropped from almost 100% to levels of 20% after the year 2000. Note that the experiment’s limitations allow us not to interpret these 20% as remnant legacy from previous use (see Material and Methods). The explained variation by the fertilisation treatment increased within the same time frame from almost 0% to values around 60%.

**Fig. 3:**
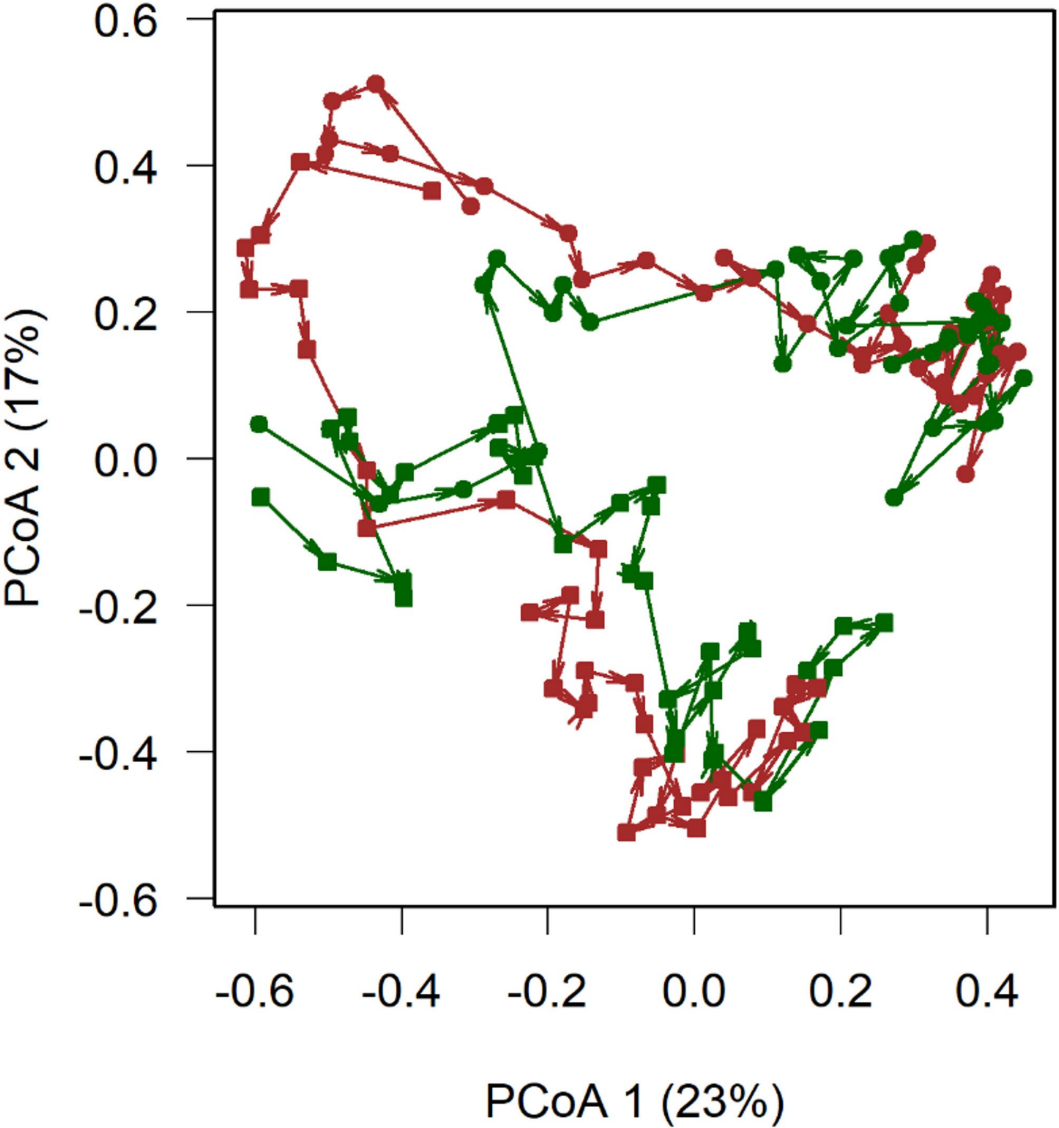
Trajectory of the succession on the four plots using a principal coordinates analysis (PCoA; explained variance of each axis is given in parenthesis). Arrows connect consecutive years for each plot. Legacy of plots: brown represents farmland, green grassland. Circle symbolizes fertilised nutrient levels and squares non-fertilised levels. Note that the trajectories show considerable variation as indicated by the variations in the angle of arrows. Overall, however, there is a clear convergence/divergence of the successional trajectories according to the fertilisation treatment.

**Fig. 4:**
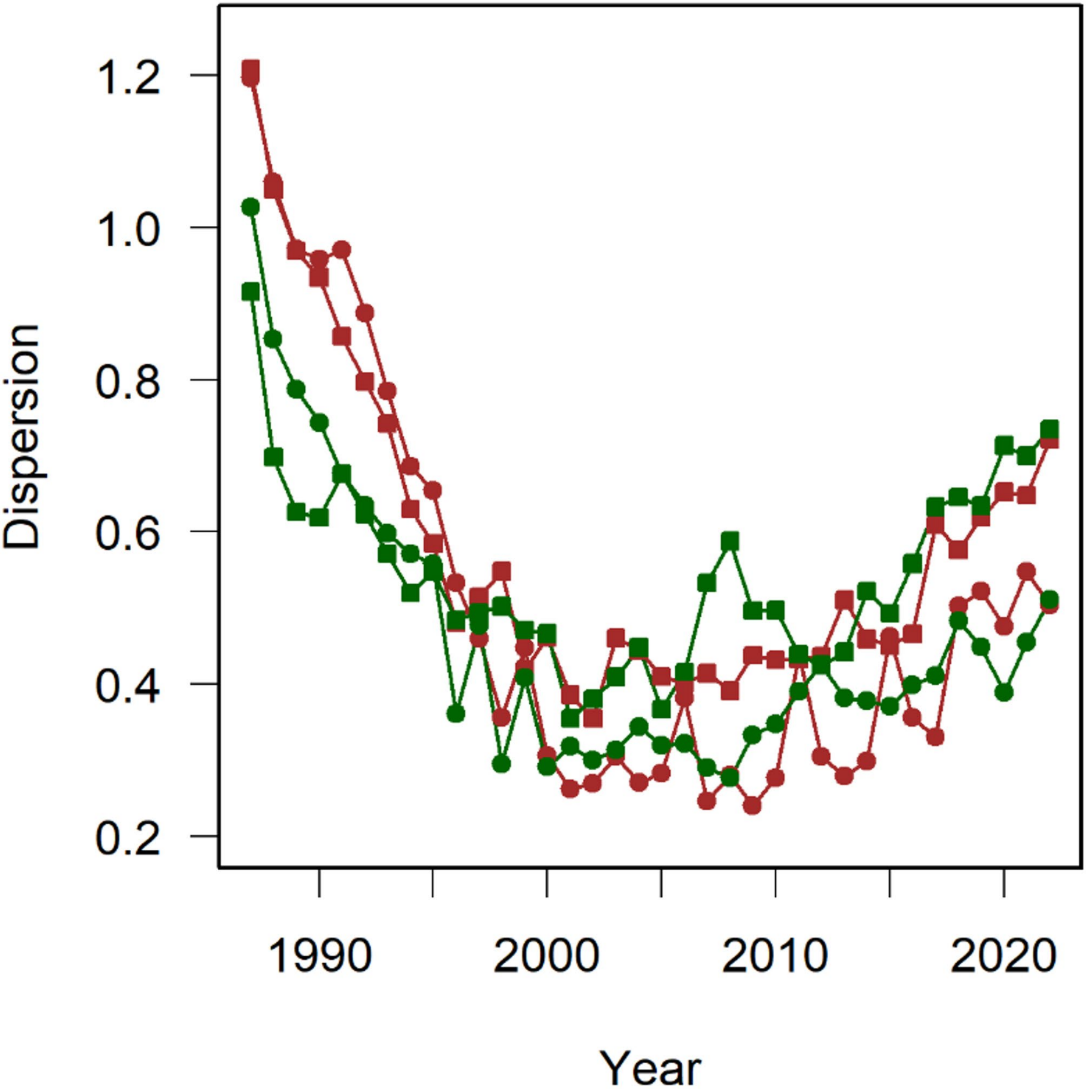
Plot of the distance of each sample from the median of the respective plot (labelled “dispersion”) versus the year of sampling. This dispersion measure is based on the first two principal coordinates of the distance matrix between samples (see Fig. 3). Note that the plots of each nutrient treatment approach its median around the year 2000. After this year, distances remained constant. After a further ten years, the distances increased again in all four plots. Symbol colour indicates the legacy of plots: brown represents farmland and green grassland. Circles indicate releveés on fertilised plots and squares on non-fertilised plots.

Variation in community composition explained by the legacy of land use decreased (Fig. 2a), whereas variance explained by the nutrient treatment increased from almost 0–60% after the year 2000 (Fig. 2b). Richness and explained variance reached an upper limit after the year 2000. The plot of a principal coordinate analysis of the distances showed clearly that the plots with similar fertilisation treatments converged to plant communities with a certain similarity in species composition (Fig. 3). Correlation between the trajectories of plots that differ in the fertilisation treatment showed significant positive correlations indicating divergence, whereas the trajectories of plots with the same fertilization treatment showed significant negative correlations indicating convergence (Table 2). The arrows in Fig. 3 often showed changes in the direction, indicating that there was still unexplained variation in species composition of communities after having reached a stable state. As noted above, the angles should not be correlated across plots if these variations are random. We found a mixed result with some significant correlations (Table 3). Plotting the distance of each year from the community median of the respective plot (in Fig. 4 labelled “dispersion”) showed a clear decrease with time. It levelled off again after the year 2000. However, this graph also shows an increase in the distance from the community median after 2010 leading to parabolic patterns (Fig. 4). A simple way to test for such a parabola is fitting a quadratic function and we used all years in Fig. 4 in the test. The resulting regression using all four plots leads to a significant and positive estimate for the quadratic term of the independent variable year indicating a parabola opening upwards. This indicates that, in recent years, the communities have started diverging from their median again. Overall, Fig. 4 suggests that the stable state reached between 2001 and 2010 is transient.

For the indicator species analysis, we used in response to the conclusions from Fig. 4 the period 2001 to 2010. Five species characterized the fertilised plots, and 29 characterized the non-fertilised plots (Table 4). Furthermore, and as expected, the species characterizing the fertilised treatments had, on average, a higher N-value compared to the few species that characterized the two non-fertilised plots (Table 4). We additionally performed an indictor value analysis for the period 2011 to 2022, where our data suggested a development leaving the transient stable state reached before. But the results were similar to the previous period, with four species as indicators for fertilised plots and 34 species as indicators for non-fertilised species plots with a considerable overlap of species.

**Table 1:** Correlation of the species richness on each plot with time, mean richness and range of species richness using only richness values from the years 2001 to 2022. Note that none of the four correlation coefficients was significant and that fertilisation leads to a reduction of mean species richness despite considerable overlap in the ranges of species richness (see also Fig. 1b).

	*r*	*P*	Mean richness	Range richness
Farmland/fertilised	-0.17	0.32	25	18 - 35
Farmland/non-fertilised	0.07	0.67	41	27 - 57
Grassland/fertilised	0.25	0.34	23	17 - 29
Grassland/non-fertilised	0.31	0.31	38	29 - 49

**Table 2:** Matrix of Mann-Kendall Tau statistics of the convergence/divergence test between the trajectories of the four plots labelled according to their previous use as well as for the experimental treatment (fertilised and non-fertilised). Negative values indicate convergence, and positive values indicate divergence. Below the diagonal, P-values are given. Significant values of the test statistic are in bold. For details on the statistical approach, see^36^.

	Farmland		Grassland	
	Fertilised	Non-fertilised	Fertilised	Non-fertilised
Farmland/fertilised		**0.53**	**-0.48**	-0.17
Farmland/non-fertilised	< 0.001		0.08	**-0.30**
Grassland/fertilised	< 0.001	0.52		**0.71**
Grassland/non-fertilised	0.15	0.017	< 0.001	

**Table 3:** Matrix of Mann-Kendall Tau statistics for the correlation of the angles between consecutive years of vegetation samples for four plots labelled according to their previous use and for the experimental treatment (fertilised, non-fertilised). Below the diagonal P-values for the statistics are given. Significant values of the test statistic are in bold. We also list a measure of the directionality of the whole trajectory for each plot. For details on the statistics see^36^.

	Farmland		Grassland	
	Fertilised	Non-fertilised	Fertilised	Non-fertilised
Farmland/fertilised		**0.30**	**0.29**	0.21
Farmland/non-fertilised	0.011		0.077	**0.54**
Grassland/fertilised	0.017	0.53		0.16
Grassland/non-fertilised	0.089	< 0.001	0.18	
Directionality	0.45	0.43	0.44	0.43

**Table 4:** Indicator species analysis for plots with different nutrient levels using the releveés from 2001 to 2010. Species were selected as indicator species if P was ≤ 0.05. If available, we also note the Ellenberg N indicator values extracted for each species from the *BiolFlor* database^38^ between the two groups of the nutrient treatment, the mean N indicator values were significantly different (*P* = 0.02; linear model; not corrected for phylogeny).

	Value	P	N indicator value
Fertilised plots – 5 species			
1. *Carduus acanthoides*	0.769	0.005	7
2. *Atriplex oblongifolia*	0.758	0.005	6
3. *Viola arvensis*	0.709	0.020	
4. *Linaria vulgaris*	0.671	0.010	5
5. *Silene latifolia*	0.632	0.005	7
			Mean = 6.3
Non-fertilised plots – 29 species			
1. *Prunus mahaleb*	0.999	0.005	2
2. *Rumex thyrsiflorus*	0.997	0.005	4
3. *Achillea millefolium*	0.988	0.005	5
4. *Rosa canina*	0.972	0.005	
5. *Pastinaca sativa*	0.947	0.005	5
6. *Hypericum perforatum*	0.893	0.005	4
7. *Vicia tetrasperma*	0.892	0.005	5
8. *Euonymus europaea*	0.803	0.005	5
9. *Vicia hirsuta*	0.763	0.005	4
10. *Epilobium tetragonum*	0.742	0.005	5
11. *Acer tataricum*	0.739	0.005	
12. *Ligustrum vulgare*	0.733	0.005	3
13. *Amorpha fruticosa*	0.707	0.005	
14. *Cornus sanguinea*	0.707	0.005	
15. *Symphoricarpos orbiculatus*	0.707	0.005	
16. *Deschampsia cespitosa*	0.698	0.005	3
17. *Poa nemoralis*	0.671	0.005	4
18. *Vicia villosa*	0.657	0.030	5
19. *Poa angustifolia*	0.652	0.040	3
20. *Hieracium sabaudum*	0.632	0.005	2
21. *Malus domestica*	0.632	0.005	6
22. *Torilis japonica*	0.632	0.015	8
23. *Crataegus laevigata*	0.629	0.005	5
24. *Vicia sativa*	0.614	0.010	
25. *Cerastium arvense*	0.592	0.005	4
26. *Rubus caesius*	0.592	0.015	7
27. *Cerastium holosteoides*	0.548	0.040	5
28. *Daucus carota*	0.548	0.010	4
29. *Galium verum*	0.538	0.035	3
			Mean = 4.4

## Discussion

Our experiment has some clear drawbacks. First of all, the legacy of land use is not replicated. Thus, we cannot interpret the variation in species composition between the two land-use types. The variation in species composition and abundance structure between these two types of plots may be due to the legacy of land use, but also to differences in the environmental conditions between the plots. Second, we have only two plots for each treatment. This is a small sample size compared to other studies. Nevertheless, our results support sophisticated studies with more replicates^2^. Furthermore, our experiment provides independent support for the resource-dependent coexistence of species from continental Europe. A recent analysis used an experiment in North America^2^, an area with an evolutionary history differing from Europe. Europe is compared to North America characterised by a long history of agricultural use by humans. Such differences in the selection regime might have fostered the evolution of adaptive syndromes that differ between the two continents. Thus, our results also suggest that the resource-dependent coexistence of species is a general process influencing the structure of plant communities.

Our results support the three hypotheses listed in the introduction. First, we found that communities became stable after more than ten years. This is supported by the analyses of species richness, explained variance by the nutrient treatment and community trajectories. The analysis of species richness provides in this respect the least convincing result. A stable species richness might also indicate a dynamic equilibrium with changing species composition, similar to the dynamic equilibrium predicted by the island biogeography. Nevertheless, the time span between the start of the experiment and reaching a stable state is the same for all variables that allow to estimate this period. Second, the species composition of the stable states differed between the two fertilisation treatments. Third, our results were in agreement with the predicted difference in Ellenberg´s N indicator values between species typical for the surveys on fertilised and non-fertilised plots.

Our data, however, point to some further details. First, succession needs up to 15 years to reach a stable state. Natural processes in plant communities have a long time frame, even for herbaceous communities^2,3^. When evaluating management projects and projects concerned with restoration ecology, we cannot always expect the predicted results to appear within the lifetime of such projects. Second, the stable state was transient. During the last 10 years communities started to change again in species composition and abundance structure, although less dramatic compared during first years of the succession. These recent changes are not an effect of the establishment of phanerophytes or long-lived species (Supplementary information: Fig. S2). We can only speculate about the reasons. For example, the inflow of propagules from outside might drive the plant communities away from the local steady-state. This inflow of propagules might differ considerably between species and these “slow” propagules might represent species with a more widespread occurrence of plant species. This species pool might represent the potential natural vegetation and/or might also represent the response to climate warming. However, the dynamics of phanerophytes suggest that the cover-abundance of trees is still increasing (Supplemental information Fig. S2) contributing on all plots to a change in community composition. With our limited data set, we are not able to analyse these new developments in detail; we are only able to note that these new and slow changes are independent of the fertilisation treatment and indicate that other factors besides the nutrient supply influence plant communities.

The result that resource-based coexistence and not priority effects drive the succession of the communities is not a real surprise. Secondary succession is also triggered by the seed bank^39,40^. Species with small, roundish seeds tend to produce a dense and persistent seed bank^41,42^. This might be only a small fraction of species. Nevertheless, the seed bank is to some extent a memory that has saved previous states of the system^40^. If the germination of the seeds needs special conditions, it might take some time until these species succeed to establish in the communities.

We found considerable unexplained variation in our trajectories of the plant community composition across years as indicated by variations in the angle of trajectories connecting consecutive years (Fig. 3). Another indication is the residual variation of the explained variation by land-use legacy and fertilisation presented in Fig. 2. Fertilization treatment after the year 2000 explained around 57% of the variation, and the plot legacy (or associated environmental factors) 25% (Fig. 2). Therefore, on average 18% of the variation across the four community matrices of each year remained unexplained. Again, we regard this unexplained variation as a sign of additional processes influencing succession (e.g., weather conditions, random recruitment, seed bank).

Further evidence of a random component during succession comes from analysing the trajectory angles between consecutive vegetation samples (Table 3). Correlated angles between plots might indicate some predictable influence on community dynamics, e.g. weather conditions. In contrast, the absence of correlation between angles of the trajectories between consecutive years indicates random processes. The found correlations are comparatively low, suggesting that there is an important random variation of species composition from year to year after having reached a stable state. Overall, the low but sometimes significant correlations (Table 3) suggest that all these possibilities, including random arrivals, are at work and influence the trajectories. We have not correlated these angles with weather conditions. With the many possible weather variables, the various time spans between weather events and their effect on plant communities, a correction of the P-value is needed to consider an effect significant. With the small number of experimental plots and the temporal autocorrelation, this leads to non-convincing results.

The transient communities reached after ten to 15 years differed in species richness, with fewer species on the fertilised plots, at least for the grassland. Besides nutrients, the soil pH is an important determinant of species richness. Species richness declines rapidly below a pH of 4.5^43^. Fertilisation might lead to acidification depending on the type of fertiliser^44^. For the experiment, calcium ammonium nitrate was used. The Ca of this fertiliser seems to mitigate acidification^45^. To check for the possibility of acidification, we used the Ellenberg R indicator values. We found no decrease in the R values averaged across recorded species when comparing the values between the first (1987) and last community sample (in 2022) of each plot: mean R value of farmland fertilised: 6.9 (1987) versus 6.8 (2022); farmland non-fertilised: 7.1 versus 7.2; grassland fertilised 6.6 versus 7.1; farmland non-fertilised 6.6 versus 7.1.

However, a comparison of quantitative estimates of the reduction in species richness by fertilisation with the Park Grass Experiment in Rothamsted leads to similar estimates (see Fig. 3 in^43^ for the Park Grass Experiment). For Rothamsted it is reported that adding 50 kg N ha^− 1^ leads to a reduction of 6.5 plant species using the results of a multivariate model^30,43^. In our experiment, 120 kg N ha^− 1^ were added, and thus, from the Park Grass Experiment in Rothamsted, one would predict a decrease of 15 plant species for our system. We found a decrease in species richness between 10 and 20 species using the data in Fig. 1b. Such a match of prediction and experimental results from two areas with different environmental conditions and species pools is unusual. It underlines the importance of resource availability for co-existence patterns. Note also that the number of species characterising fertilised plots is much lower than the number of species characterising non-fertilised plots.

This reduction of plant species richness with an increase of nutrient supply is well-known from observational and experimental studies^30,43,46^: systems with a high nutrient supply show a decrease in species richness. These experiments and numerous observational studies showed that the inflow of nutrients (e.g. nitrogen via the air and the intensification of agriculture) leads to a decrease in the diversity of plants. Plants form the basis of numerous biotic interactions with higher trophic levels (herbivorous insects) or mutualists (pollinators). Consequently, the inflow of nutrients also decreases the abundance and diversity of associated insects^47,48^. Furthermore, land-use intensification is among the primary drivers of biotic homogenization across broader spatial scales^49^. Based on our experiment and the available literature, we want to reiterate the need to control the influx of nutrients into the environment to conserve biodiversity.

## Supplementary Information

Below is the link to the electronic supplementary material.

Supplementary Material 1

## Data Availability

The datasets generated and/or analysed during the current study are not publicly available due to the fact that they are used for a further analysis but are available from the corresponding author on reasonable request.
