# Supplementary material for: Plant communities converge to resource-dependent transient states during succession on old fields
Source: Sci Rep. 2025 Aug 23;15:31070. doi: 10.1038/s41598-025-16501-8 (PMC12374994; doi:10.1038/s41598-025-16501-8)
Supplement: Supplementary file 1 — Supplementary Material 1 [file 41598_2025_16501_MOESM1_ESM.docx]

**Supplementary information**

**Plant communities converge to resource-dependent transient states during succession on old fields.**

Jutta Stadler^1^, Roland Brandl², Stefan Klotz^1^

1: Department of Community Ecology, Helmholtz Centre for Environmental Research- UFZ, Theodor-Lieser-Str. 4, 06120 Halle/Saale, Germany

2: (retired) Faculty of Biology, Department of Ecology, Philipps-Universität Marburg, Karl-von-Frisch Str 8, 35032 Marburg, Germany; private address: Weikenreuth 1, 9500 Heinersreuth, Germany

**Mail of the first author:** jutta.stadler@ufz.de

Supplementary information:

Stadler et al:

Plant communities converge to resource-dependent transient states during succession on old fields.

Weather and climate:


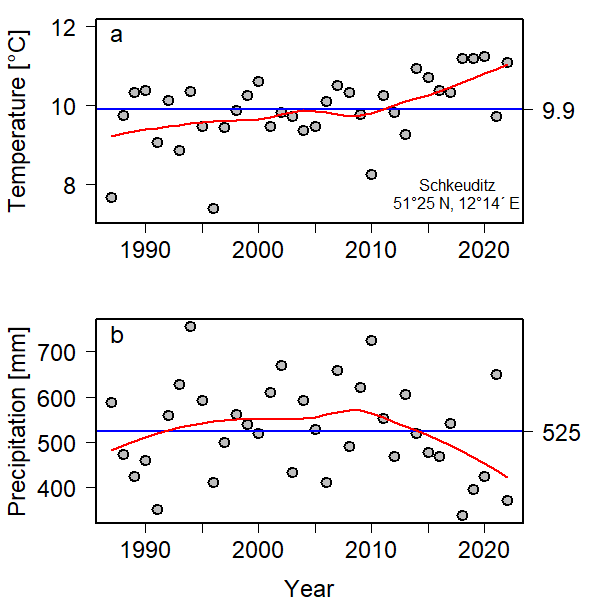


**Fig. S1:** Mean annual temperature as well as yearly precipitation of the nearby official weather station in Schkeuditz for the analysed time span of the experiment from 1987 to 2022. The blue line represents the mean for the observational period, while the red line shows a smoothed fit obtained using local polynomial regression with the loess function in R, using standard values for span and degree. The yearly temperature averaged across the years from 1934 to 2024 was 9.4 °C with a mean precipitation of 534 mm (station Leipzig Schkeuditz: N 51°26´ E 14° 14´). Note that, compared to this period, the weather over the last 36 years has been 0.5 °C warmer and slightly drier. Note also the increase of mean annual temperature during the sampling period (r = 0.49, P = 0.002)

Supplementary information:

Stadler et al:

Plant communities converge to resource-dependent transient states during succession on old fields.

Life forms:


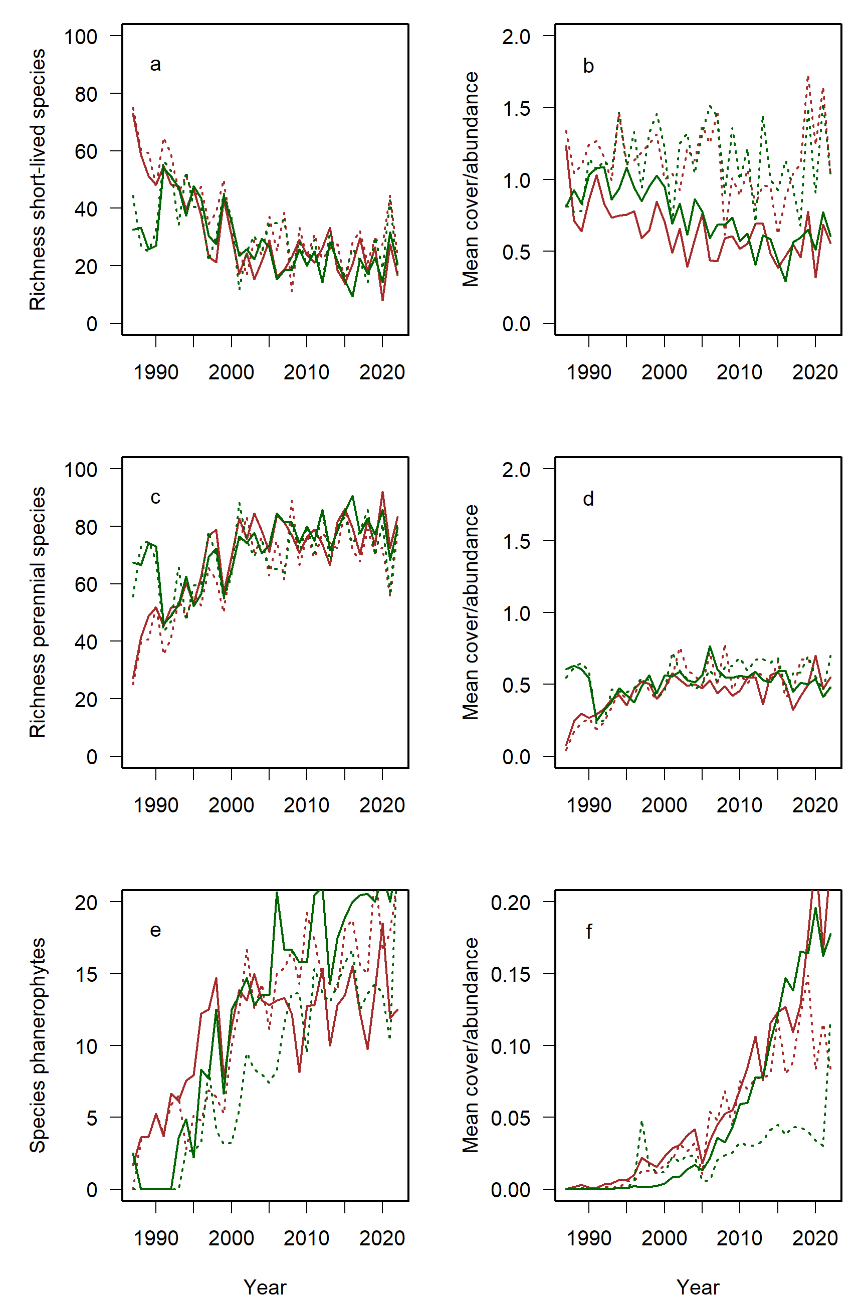


**Fig. S2:** Dynamics of life forms during the succession in Zöberitz. We analysed percentages of species richness (left column) as well as mean cover abundance (right column). (a, b) Short-lived species are defined as species living either one or two years. (c, d) perennial species that live at least 3 years and (e, f) phanerophytes. The information was extracted for each species from the *BiolFlor* database [1]. Solid lines indicate fertilised and dotted lines non-fertilised plots. The colour indicates the land-use legacy, with brown lines representing farmland and green lines representing grassland.

In general, the observed changes in the percentage of species on plots and mean cover-abundance followed the well-known patterns of succession on abandoned land [2,3]. For short-lived species, we found higher values of mean-cover abundance for non-fertilised plots. We have no hypotheses to interpret this difference, and therefore, we ignored this observation in the main text.

**References**

1. Klotz, S., Kühn, I. & Durka, W. *BIOLFLOR*: Eine Datenbank mit biologisch-ökologischen Merkmalen zur Flora von Deutschland. Schriftenreihe für Vegegtaionskunde **38** (2002).
2. Prévosto, B. *et al.* Impacts of land abandonment on vegetation: successional pathways in European habitats. *Folia Geobotanica* **46**, 303-325 (2011).
3. Stadler, J., Klotz, S., Brandl, R. & Knapp, S. Species richness and phylogenetic structure in plant communities: 20 years of succession. *Web Ecology* **17**, 37-46 (2017).
